# Evaluation of School Lunch Quality in Accordance with the Quality Standard for Meals in Schools of the German Nutrition Society in a Selection of Thuringian Schools

**DOI:** 10.3390/nu18091424

**Published:** 2026-04-30

**Authors:** Nadine Knutti, Ulrike Trautvetter, Sara Ramminger, Theresa Pörschmann, Stefan Lorkowski, Manja Andreß

**Affiliations:** 1 German Nutrition Society—Section Thuringia, 07743 Jena, Germany; 2 Institute of Nutritional Sciences, Friedrich Schiller University Jena, 07743 Jena, Germany; 3 SRH University of Applied Sciences Heidelberg Campus Gera, 07548 Gera, Germany

**Keywords:** school meals, nutrients, quality standard, lunch, DGE/ÖGE, reference value

## Abstract

**Background:** School meals are crucial for children’s development and can contribute to the prevention, amongst others, of obesity and type 2 diabetes mellitus. We analyzed the conformity of meal composition with the quality standard for meals in schools (QST) of the German Nutrition Society (DGE) and reference values for nutrient intake of the nutrition societies of Germany and Austria (DGE/ÖGE) in Thuringian schools. **Methods:** Components of the school meals (portions in primary and secondary schools) were collected at two timepoints (T1 and T2). The contents of selected nutrients (protein, carbohydrates, fat, fatty acids, dietary fiber, vitamins, minerals) were analyzed and tested for alignment with the aforementioned adapted reference values. **Results:** More than half of the school meals examined were ovo-lacto-vegetarian meals (T1: 64%; T2: 63%). The energy content of macronutrients and the contents of vitamins B_1_ and E, folate, calcium, iron and magnesium covered the requirements of the DGE/ÖGE reference values. Good *n*-6/*n*-3 PUFA ratios between 2.6 and 4.1 were found. In contrast, vitamin C was not detectable in 88.5% (T1) and 90.6% (T2) of the tested meal components, and sodium references were exceeded by a factor of five to eight. Additionally, the total sugar content of the meals tended to be high, with 85% of all meals exceeding the lower energy limit for free sugars (≥7 to <10 years) and >70% exceeding the upper limit (≥10 to <19 years) set forth by WHO and DGE/ÖGE. **Conclusions:** In the process of school meal preparation, attention should be paid to the preservation of vitamin C and the economical use of salt and sugar.

## 1. Introduction

A healthy and sustainable diet is crucial to promote the physical and mental development of children and adolescents. School meals play a central role in the prevention of diet-related diseases such as type 2 diabetes mellitus or obesity in children and adolescents. According to the World Health Organization (WHO), occurrence of obesity early in life is substantially increasing or stabilizing at very high levels in almost all European countries [1]. In the KIGGS study (2014 to 2017, wave 2) with 15.023 participants, body height and weight of children and adolescents (3–17 years) in Germany were analyzed, resulting in a high prevalence of 15.4% and 5.9% for overweight and obesity, respectively [2]. Self-rating of the individual health in children and adolescents and health rating by their parents, as well as overweight and obesity in children and adolescents, still strongly depend on the socioeconomic status of the family [2,3,4].

Currently, 86.8% of pupils have the opportunity of a warm school meal in Germany [5], but actual participation rates are much lower [5]. As described in the literature, school meal programs have been shown to play a pivotal role in the reduction of non-communicable diet-related diseases in adulthood through the provision of healthy meals to a more extensive group of children [6]. It can be assumed that public subsidization of healthy school food facilitates greater access to such meals and thus constitutes a vital instrument in ensuring that children and adolescents from diverse social backgrounds receive the recommended nutrients for optimal physical and mental development, and to prevent diet-related disorders.

The reference values of the German and Austrian Nutrition Societies (DGE/ÖGE reference values) define different nutrient and energy intakes for good health depending on age, sex and specific phases of life (e.g., pregnancy) [7]. The nutrient requirement for optimal human growth and development results in divergent reference values between children, adolescents and adults. Besides these impact factors, the total energy intake depends on physical activity. To reach nutrient DGE/ÖGE reference values for nutrient intake for pupils of primary level (age 7 to <10 years) and of secondary level (age 10 to <19 years), the quality standard for meals in schools (QST) of the German Nutrition Society (DGE, Deutsche Gesellschaft für Ernährung) defines a physical activity level (PAL) of 1.4 for menu planning in mass catering [8]. Consequently, the fat intake of children and adolescents should constitute between 30% and 35%, and carbohydrates should provide >50% of the total energy intake [8]. An optimal protein intake is set at 0.8–0.9g/kg body weight per day, resulting in 26 to 62 g protein per day in the aforementioned age groups, based on normal body weight [9]. Besides the energy-supplying nutrients, a wholesome diet for children and adolescents requires a balanced intake of vitamins, minerals, trace elements, dietary fiber and phytochemicals [8]. In addition, high intakes of fat, sugar, salt or sodium have adverse effects on the health of children and adolescents and are the subject of health campaigns in several European countries [10]. Accordingly, the DGE recommends the controlled use of these food components; e.g., free sugars should contribute no more than 10% of total food energy due to their contribution to body weight and dental caries prevalence in children and adolescents [11].

The QST is based on the DGE nutrition circle and the DGE three-dimensional food pyramid [8]. The QST defines food quality and frequency of different food groups, such as grain, grain products and potatoes, vegetables and salad, fruit, milk and dairy products, meat, sausage, fish and eggs and oils and fats to facilitate nutrient-optimized menus appropriate for all age groups of pupils [8]. According to legal requirements of Thuringia, school lunches must meet current nutritional standards for a balanced, age-appropriate, wholesome and healthy school lunch in Thuringia [12]. The QST meets these requirements. By 2030, the federal government aims to make the QST compulsory in schools across Germany [13].

The present results were obtained in the course of the German (Thuringian) project on the quality of school lunch meals “(K)eine Frage des Preises? Projekt zur Teilsubventionierung der Mittagsmahlzeiten zur Qualitätsverbesserung der Speisenversorgung an Thüringer Schulen” (TSMP). In this project, meal providers got a partial subsidy for preparation of school lunch meals. The precondition was to adjust the lunch meal to the QST. The partial subsidy was paid out to the school authorities, who passed the subsidy to the respective meal provider of the project school. Furthermore, school authorities were able to get subsidies for the respective project school for kitchen appliances to reduce the time the food had to keep warm, as well as furnishings to improve the ambiance in the canteen/dining hall. The project was financed by the Thuringian Ministry of Migration, Justice and Consumer Protection in the time from 2018 until 2019 (funding period 1) and from 2020 until 2024 (funding period 2). The following results refer to funding period two. The aim of the present study was to evaluate the compliance of the actual nutrient content of school meals with the QST. To this end, meal components were collected in Thuringian project schools, followed by a nutrient analysis to quantify compliance with the adapted requirements of the DGE/ÖGE reference values according to the so-called “quarter approach” [7,8].

## 2. Materials and Methods

### 2.1. Study Characteristics

The study consisted of two separate sample collections. The included school counts and types and the count of analyzed components and meals, as well as the types of food preparation/serving, are listed in Table 1.

### 2.2. Sample Collection

For the laboratory analyses of nutrient contents and caloric values, individual food components were collected from school lunch meals, considering planned portion sizes. Samples were collected after serving the meal to the last guest. Thereby, individual food components of each meal (such as potatoes, carrots, gravy, dessert, salad, etc.) were weighted with a digital balance (Soehnle, Nassau, Germany, scaling/resolution: 1 g). Afterwards, each component was homogenized separately by an immersion blender before it was transferred into labeled, pre-cooled (−20 °C) sample storage containers. Components were frozen immediately and stored until analysis at −20 °C. The process of sample collection was conducted in a consecutive manner at two different timepoints (T1: September 2020 to November 2021 and T2: January 2022 to December 2022) to achieve a more comprehensive insight into the quality of school meals. During the pandemic (COVID-19) in 2021, a series of sample collections were conducted by school staff after serving the meal to the last guest. The samples collected were subsequently delivered to the study staff without any personal contact. The samples were then treated as described above. Regarding the underlying project, it is important to emphasize that between sample collections, meal providers worked continuously to improve the quality of catering in accordance with the QST in the participating schools.

### 2.3. Laboratory Analyses of Nutrient and Energy Content

The composition of collected food components in lunch meals at both timepoints (T1, T2) was analyzed in an external certified laboratory (SGS Analytics, Jena, Germany). Methods for macro- and micronutrient quantification by the external laboratory were as follows: Total protein was analyzed via the Kjeldahl method, total fat content by acid hydrolysis (HCl) followed by gravimetry and Soxhletextraction (hexane), carbohydrates (glucose, fructose, saccharose, lactose, galactose, maltose, starch) were calculated from single component quantification and fiber content quantification was based on a gravimetry–enzymatic assay. The energy content was calculated from the individual energy values of the constituting macronutrients. Liquid chromatography mass spectrometry was used for the quantification of folate (IKB 00.12.20.LC: 2020), and high-performance liquid chromatography for vitamin B_1_ (DIN EN 14122:2014-08) and vitamin E (DIN EN 12822:2014-08). Analysis of vitamin C content was performed with a colorimetric/enzymatic (tetrazolium salt MTT, ascorbate oxidase) assay (R-Biopharm, Darmstadt, Germany). For the quantification of the mineral content (calcium, iron, magnesium, sodium), the initial decomposition was followed by inductively coupled plasma–mass spectrometry (ASU § 64 LFGB L00.00-19/1, DIN EN 13805:2014-12 and DIN EN ISO 17294: 2017-01).

Fatty acid composition was analyzed in homogenized food components with at least 1% fat content. For this, total lipids were extracted from 1.0 to 2.0 g of the homogenized samples with methanol/chloroform/2% sodium chloride (ratio 1:2:1 (*v*/*v*/*v*)). These lipid extracts were transmethylated using sodium methylate. Subsequently, fatty acid methyl esters (FAMEs) were dissolved in n-hexane for analysis. FAME separation was performed using gas chromatography (GC-2030, Shimadzu, Duisburg, Germany) equipped with a cooled autosampler and flame ionization detector. A medium-polarity fused silica capillary column was used (DB-225MS, 60 m × 0.25 mm i.d. with a 0.25 mm film thickness; Agilent Technologies, Waldbronn, Germany). The initial oven temperature was held at 70 °C for 2 min, then increased to 180 °C (by 10 °C/min), further increased to 220 °C (by 2 °C/min) and held at this temperature for 5 min. Afterwards, it was increased to 230 °C (by 2 °C/min) and held for 15 min. The injector and detector temperatures were kept constant at 260 °C. Hydrogen was used as a carrier gas. To identify fatty acid peaks, different reference standards were used as FAME mix: No. 463, 674, (NU-CHEK PREP, Elysian, MN, USA), BR2, BR4, ME 93 (Larodan, Solna, Sweden), and PUFA No. 3 (Supelco, Bellefonte, PA, USA). For peak integration, Lab Solutions software for GC version 5.93 (Shimadzu, Duisburg, Germany) was used.

### 2.4. Data Analysis

In general, the presented data represents the macro- and micronutrient content of the food components offered to children and adolescents via the school lunch meals. The meal providers communicated portion sizes and general composition of the lunch meals to the project team. Analyzed individual food components were adjusted according to this information to achieve final meal composition served at schools (Table 1).

Lunch meals were designated as “ovo-lacto-vegetarian meals” when no meat or fish components were included; milk, dairy products and eggs were counted as ovo-lacto-vegetarian components. Depending on their size, the lunch meals were divided into “primary portion” for pupils aged between 7 and 10 years (smaller portion size) and “secondary portion” served to pupils aged between 10 and 19 years (larger portion size).

The energy references for lunch meals were derived from the DGE/ÖGE recommendations for daily energy intake for the age groups ≥ 7 to <10 years (primary portion) and ≥10 to <19 years (secondary portion) with a PAL value of 1.4. In accordance with the QST, the consumption of lunch should comprise 25% of the total daily energy intake (“quarter approach”) [7,8]. The mean value was calculated from the resulting energy intake for both sexes (female, male). The energy density (ED) of food was calculated in kcal/g as the energy content (in kcal) per unit of food weight (g or 100 g) [14]. The calculated energy contribution of the main nutrients (carbohydrates, fat, proteins and dietary fiber) to the total daily dietary energy was defined as energy percentage (E%).

To evaluate the measured macro- and micronutrient contents and frequencies in meals, the QST and DGE/ÖGE reference values for the age groups ≥ 7 to <10 years and ≥10 to <19 years were considered and adapted to the requirements of the lunch meal proportion (“quarter approach”) [7,8]. Thereby, reference values for the age group ≥10 to <19 years were calculated by the mean of reference values of three age groups, i.e., ≥10 to <13 years, ≥13 to <15 years and ≥15 to <19 years. Reference values for protein intake of children and adolescents were considered according to Richter et al. [9]. Salt content of food components was calculated from the sodium content (× 2.54) according to the EU regulations 1169/2011 and the WHO conversion indication [15].

Statistical analysis was performed using Microsoft Excel 365 (Redmond, WA, USA), as well as the statistical software package IBM SPSS Statistics 29.0.2.0. (IBM Deutschland, Böblingen, Germany). A descriptive statistical analysis was performed (mean, standard deviation and 95% confidence intervals (CI, 95%)) for energy, ED, and E%, as well as selected macro- and micronutrients in school meals.

## 3. Results

In order to evaluate the quality of the school meals in accordance with the adapted reference values of the DGE/ÖGE (“quarter approach”), food samples resulting from two different collection timepoints (T1, T2) were analyzed for their composition, a variety of macro- and micronutrients and energy contents.

### 3.1. General Meal Composition

**Figure 1:**
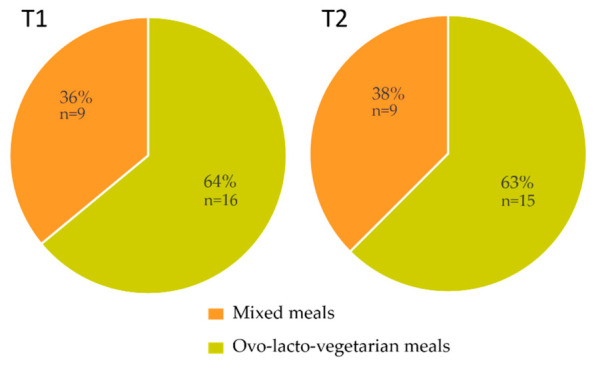
Proportions of mixed and ovo-lacto-vegetarian meals in schools at both timepoints. Timepoint T1, *n* = 25; timepoint T2, *n* = 24, due to rounding, some numbers may not sum exactly to 100.

At both timepoints of sample collection, at least two-thirds of the total meal count were ovo-lacto-vegetarian meals (Figure 1).

**Figure 2:**
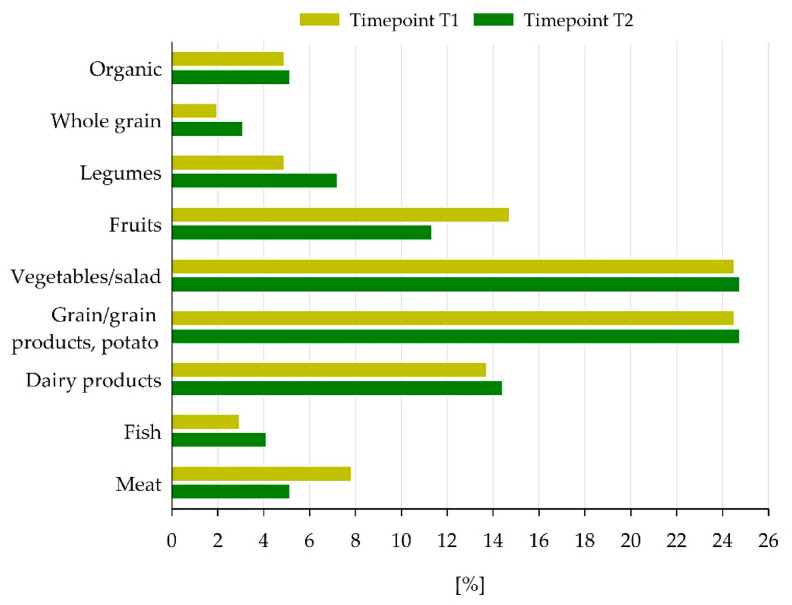
Proportion of selected food groups in relation to all food components that were examined at timepoints T1 and T2. Food groups were taken from the quality standard for meals in schools of the German Nutrition Society [8].

To preserve the health of pupils and to provide an optimal food choice, the QST describes several relevant food groups [8]. At both timepoints, the groups of vegetables and/or salads, as well as grain/grain products/potatoes, were included in all tested meals and constituted around 25% of all food compounds, respectively. Higher amounts of legumes, fish, whole grain and dairy products were apparent in T2 compared to T1. Otherwise, meat and fruits were less apparent in T2. In general, around 5% of the food components offered were of organic quality (Figure 2).

### 3.2. Energy and Energy Density (ED) of Meals

Mean meal energy (laboratory results) was generally lower in primary compared to secondary portions in T1 and T2. The mean energy content of the primary portion met the adapted requirements of the DGE/ÖGE reference values (400 kcal, PAL 1.4) at both timepoints [7,8]. The mean energy content of secondary portions was higher at T2 compared to QST criteria (517 kcal, PAL 1.4) but aligned with the DGE/ÖGE reference values criteria at T1 (Table 2) [7,8].

In both sample collections, the calculation of mean meal ED revealed similar values of 0.8 ± 0.2 (T1) and 0.9 ± 0.3 (T2) (Table 2).

To identify individual meals or food components with high energy values, their ED was categorized. An ED < 0.6 kcal/g was defined as “very low energy density” (VLED) foods/meals, whereas foods/meals with an ED > 2.5 kcal/g foods/meals or >4 kcal/g foods/meals were characterized as having a “high energy density” (HED) [16,17]. In general, food with low ED, such as vegetables, fruits or whole grains, is largely low in fat but high in fiber and/or water content. With one exception for a portion of butter in T1 (ED: 7.5 kcal/g), the ED was below 4 kcal/g for all tested meals at both timepoints, T1 and T2. However, a few components with high ED were found, such as grated cheese, fried sausage or grain products (Table 3). Very low ED below 0.6 kcal/g was apparent in nine analyzed meals, T1 (*n* = 2) and T2 (*n* = 7), and in 33 out of 96 and 33 out of 88 food components in T1 and T2, respectively.

### 3.3. Main Nutrients in Meals: Carbohydrates, Fat, Proteins and Dietary Fiber

**Figure 3:**
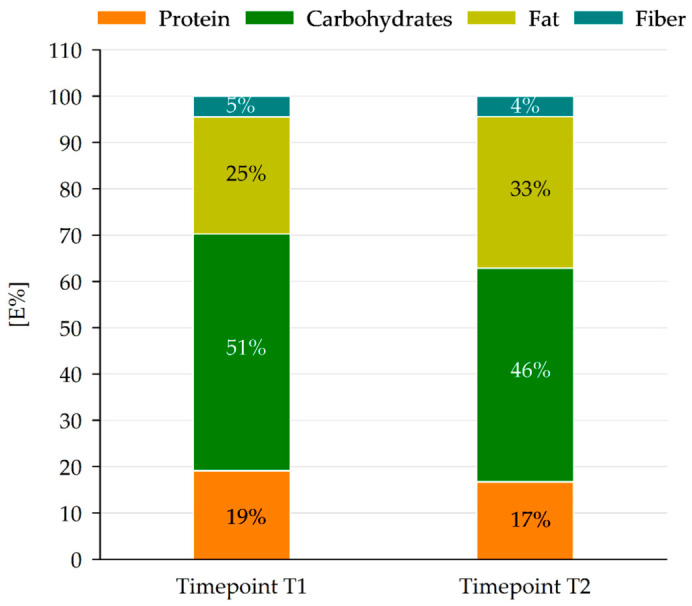
Energy contribution of the main nutrients to the total dietary energy (E%) at both timepoints. Timepoint T1 (*n* = 25), timepoint T2 (*n* = 24).

The E% of the main nutrients (carbohydrates, fat, proteins and dietary fiber) was similar in both sample collections with minor differences, especially in carbohydrate and fat contents (Figure 3).

In accordance with the DGE/ÖGE dietary energy reference values for children and adolescents [7], carbohydrates constituted around 50 E% and fat around 30 E% of the total analyzed dietary energy. Moreover, calculated CI for meal contents of carbohydrates [g] and fat [g] from laboratory analyses covered the required adapted DGE/ÖGE reference values of both age groups (Table 4) [7,8]. The protein contents are presented in detail in the following Section 3.3.1. Nevertheless, dietary fiber contents of the tested meals resulting from laboratory analysis fully met recommendations of the DGE QST in lunch meals for adults, which is in line with DGE statements on dietary fiber intake for children and adolescents [18].

#### 3.3.1. Protein

The E% of protein exceeded the adapted reference energy requirements for the primary and secondary portions, respectively (Table 4). The respective DGE/ÖGE protein reference values were exceeded by the detected mean protein contents by a factor of three for the younger pupils’ group (primary portion) and by a factor of two for the older pupils (secondary portion) (see Table 4) [7,8]. Detailed analyses of the meals focused on identifying high protein levels in the individual components. In accordance with the Nutrition and Health claims set forth by the EU, food components with protein content exceeding 20 E% of the total component energy were classified as “high in protein” [19]. Of the total meal components, 25% in T1 and 26% in T2 were “high in protein”. At timepoints T1 and T2, 14% and 18% of the ovo-lacto-vegetarian components and 11% and 8% of the animal-based components were classified as “high in protein”, respectively.

**Figure 4:**
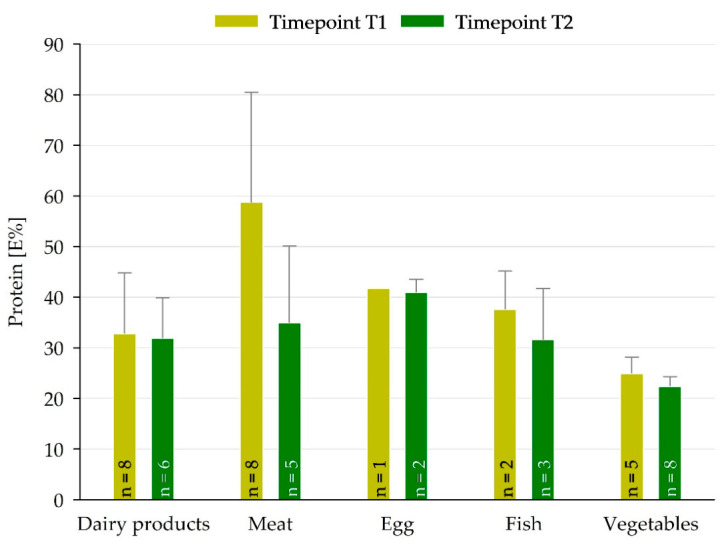
Energy contribution of protein content to the total dietary energy (E%) at both timepoints in protein-rich components, subdivided into different food categories. Mean + standard deviation.

Figure 4 shows meat, dairy and ovo-lacto-vegetarian products with the highest mean protein E% of the sample collection in different food categories. Meat and egg had the highest (>40 E%) and vegetables the lowest protein E% at both sample collection timepoints. Hence, compared to categories of animal origin (dairy products, meat, egg, fish), the vegetable category had the lowest energy content from protein (Figure 4). The difference in protein E% between the meat components at T1 and T2 may be attributed to a higher proportion of pork at T2 compared to T1, while at T1, chicken and beef were predominant.

#### 3.3.2. Sugars (Mono- and Disaccharides)

The mean sugar E% (sum of all analyzed sugars) was comparable at both timepoints, representing 18.4 ± 7.1% and 18.7 ± 9.4% at T1 and T2, respectively (sum of primary and secondary portions).

There are still no quantitative recommendations for total sugar intake of children and adolescents, as links with various diseases have not yet been established. The present results provide information about the total sugar content. The WHO issues recommendations for free sugars [20]. Thereby, free sugars are defined as mono- and disaccharides added to foods [11,20]. The distinction between natural and free sugars in the present data is not possible. Nevertheless, we classified the analyzed total sugar content of school lunch meals and meal components according to the recommendations for free sugars (<10 E% of the total energy intake) of the WHO, DGE, German Obesity Society (DAG) and German Diabetes Society (DDG) [11,20]. Hence, our calculation showed that >85% of all primary and >70% of all secondary meal portions exceeded the WHO energy limit for free sugars of max. 10 E% (Table 5). The individual high-sugar components were primarily fresh fruits, including apples or bananas, but also a variety of sweet desserts, ovo-lacto-vegetarian lasagna, marinated herring, a portion of red cabbage as a side dish and white cabbage salad in marinade.

**Figure 5:**
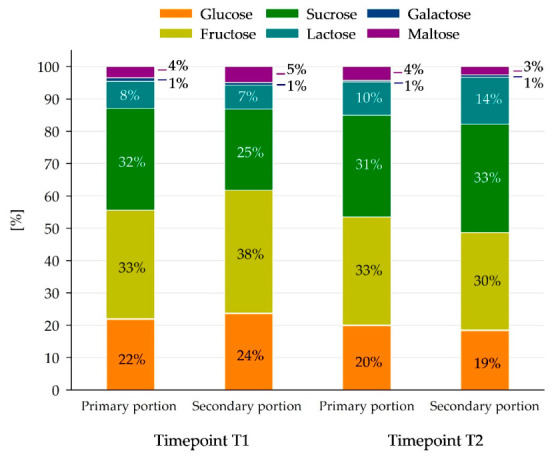
Proportion of mono- and disaccharides in the total sugar content of the dishes examined for both timepoints. Due to rounding, some numbers may not sum exactly to 100.

Figure 5 shows the profile of mono- and disaccharides with their individual contribution to the total sugar content in 100 g of the meals at T1 and T2. The most frequent sugars are fructose (around one-third), sucrose (around one-third) and glucose in primary and secondary portions. It is noteworthy that fructose contributed 6.1 ± 3.3 E% and 5.7 ± 3.5 E% in T1 and T2, respectively.

#### 3.3.3. Fat and Fatty Acid Composition

The total fat energy of all lunch meals (sum of primary and secondary) was found to be 25 E% and 33 E% at T1 and T2, respectively, which was in accordance with the DGE/ÖGE recommendations for total fat energy intake (30–35 E% of total energy intake) in children and adolescents [7].

The evidence-based guideline of the DGE on fat intake supports the DGE/ÖGE recommendations on energy proportion of saturated fatty acids (SFAs) and polyunsaturated fatty acids (PUFAs) on total energy below 10 E%, and for monounsaturated fatty acids (MUFAs), energy proportion is calculated by the difference between total fat and the sum of SFAs and PUFAs [7,21].

**Figure 6:**
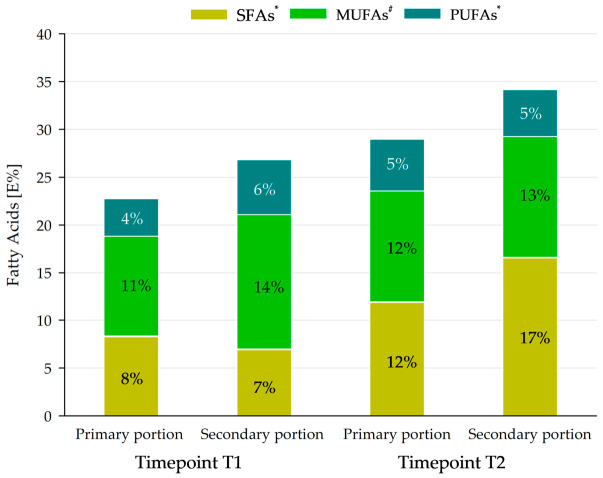
Energy contribution of saturated, monounsaturated and polyunsaturated fatty acids to the total dietary energy (E%) at timepoint T1 and timepoint T2. Primary portion: ≥7 to <10 years, secondary portion: ≥10 to 19 years, SFAs: saturated fatty acids, MUFAs: monounsaturated fatty acids, PUFAs: polyunsaturated fatty acids. *: reference values: 7–10 E% of total meal energy; ^#^: reference: 10–16 E% of total meal energy (18).

Figure 6 demonstrates that PUFA contents in primary and secondary portions were below, but MUFA contents covered the recommendations [7]. SFA contents covered or even exceeded the reference values [7]. A more detailed examination of the PUFA distribution revealed mean values of the *n*-6/*n*-3 PUFA ratio of 4.1 and 3.3 for the primary portions and 2.6 and 4.0 for the secondary portions at T1 and T2, respectively.

### 3.4. Vitamins in Meals: Vitamin C, E, B_1_ and Folate

The analyzed vitamin contents in school meals mainly covered the DGE/ÖGE and QST reference values for vitamins B_1_ and E, as well as folate, for both age groups and at both timepoints (Table 6). Vitamin B_1_ and folate slightly fell below the reference values at T1 for the younger age group (7–10 year) and also for the older pupils at T2.

**Figure 7:**
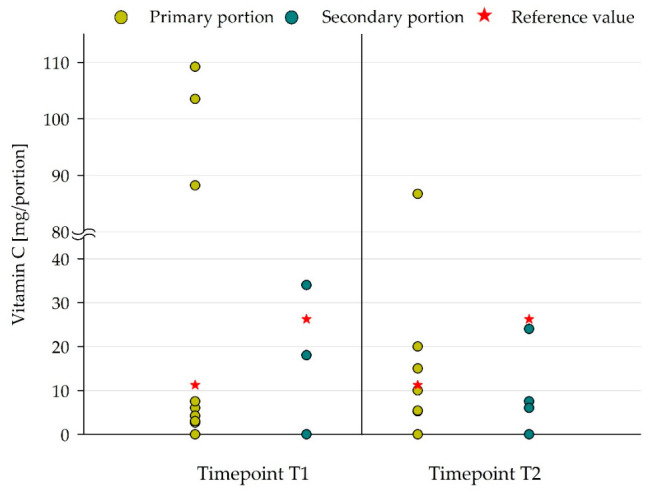
Comparison of vitamin C content of lunch meals in primary and secondary portions and associated adapted DGE/ÖGE reference values (“quarter approach”). Red star: requirements of DGE/ÖGE [7], primary portion: ≥7 to <10 years, T1: *n* = 23, T2: *n* = 21, secondary portion: ≥10 to 19 years, T1: *n* = 4, T2: *n* = 8.

The most critical vitamin in lunch meals was vitamin C (ascorbic acid). Vitamin C shows mean contents, with few exceptions, below DGE/ÖGE reference recommendations, as shown in Table 6 and Figure 7. This was mainly due to the lack of detectable amounts of vitamin C in most of the meal components, i.e., 88.5% and 90.6% in T1 and T2, respectively.

Table 7 presents a detailed view of the individual meal components contributing to vitamin C content. It is obvious that the vitamin C content of similar foods can vary greatly, such as potatoes or vegetables like paprika/tomato.

### 3.5. Minerals in Meals: Calcium, Magnesium, Iron and Sodium Chloride

Calcium, magnesium, iron and sodium were analyzed in all collected components/meals at T1 and T2. In general, the CI calculated from the measured mineral contents in the school meals covered all adapted DGE/ÖGE references (“quarter approach”) except for calcium in primary portions at T1, which fell below the reference values (see Table 8, [7,8]).

#### Sodium Content/Salt

**Figure 8:**
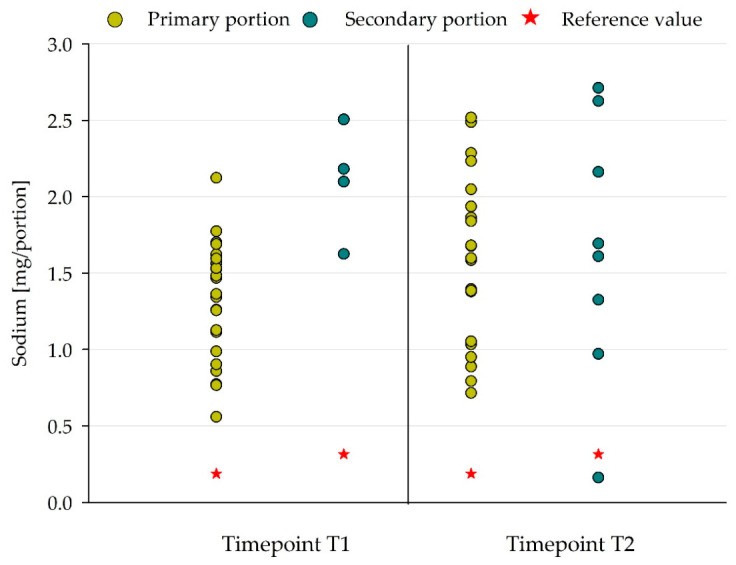
Comparison of sodium content of lunch meals in primary and secondary portions and associated adapted DGE/ÖGE reference values (“quarter approach”) [7,8]. Red star: references of DGE/ÖGE [7], primary portion: ≥7 to <10 years, T1: *n* = 23, T2: *n* = 21, secondary portion: ≥10 to 19 years, T1: *n* = 4, T2: *n* = 8.

The mean sodium contents of the meals were 1.3 g and 1.6 g per primary portion and 2.1 g and 1.7 g per secondary portion at T1 and T2, respectively. These values exceeded the adapted sodium DGE/ÖGE recommendations (“quarter approach”) on average by a factor of 7.0 and 8.5 (primary portion) and 6.7 and 5.3 (secondary portion) at T1 and T2, respectively [7,8]. Figure 8 illustrates the elevated sodium content of the lunch meals across both portion types.

To evaluate the salt (sodium chloride) content in food components and meals in relation to the WHO benchmarks for salt content in foods (mg/100 g), sodium chloride values were calculated from the measured sodium values (see chapter data analysis). The resulting mean salt contents analyzed per portion were 3.4 ± 1.0 g and 4.0 ± 1.4 g for the primary portion and 5.3 ± 0.9 g and 4.2 ± 2.2 g for the secondary portion in T1 and T2, respectively. The application of WHO salt benchmarks to the analyzed food components identified 65 (63%) and 74 (70%) food components with a salt content higher than the benchmark at T1 and T2, respectively (Table 9). It is notable that 53 out of 104 (T1) and 61 out of 106 (T2) components exceeded the WHO salt benchmark by more than 50% (Table 9).

The main component categories with sodium/salt contents above the WHO benchmark, observed in both sample collections (T1, T2), were potato and potato products, pasta/noodles/rice, processed meat, prepared salads and sauces.

## 4. Discussion

In the present study, nutrient analysis in a total of 25 (T1) and 24 (T2) meals at two different collection timepoints provides cross-sectional data and gives insights into the nutrient composition in Thuringian school meals and what ultimately ends up on the pupils’ plates. The results represent that energy and most of the investigated nutrient contents of the lunch meals fulfilled QST requirements and adapted DGE/ÖGE reference values, such as carbohydrates, dietary fiber, and vitamins B_1_ and E, as well as folate and selected minerals. Nevertheless, on the one hand, it should be considered that calculated average energy reference values adapted for lunch meals for the secondary portions comprise three age groups (10 to 13, 13 to 15 and 15 to 19 years) covering a larger energy range compared to the primary portions. From this perspective, it should be acknowledged that the energy content of lunch meals, in some cases, may be inadequate for students in higher age groups. On the other hand, a few critical nutrients, such as protein, sugar, salt and vitamin C, were identified.

The QST defines different criteria for food frequencies and quality [8]. In this regard, meat and fish frequency should not exceed one offering during a 5-day food supply, respectively [8]. This implies a mainly plant-based diet for pupils. The present study’s findings are in line with this, with most food components in both sample collections (T1, T2) being ovo-lacto-vegetarian. The count of fish and meat components was low compared to the food groups of vegetables/salads and grains/grain products/potatoes. It should be noted, however, that verification of specific QST requirements for individual food frequencies in school meals over a period of five meal days was not possible due to the study design, which comprised individual sample collections and did not include five or more replicate collections at one school. To answer this question, we examined mass catering menu plans over a defined time period (unpublished data).

To estimate the ED of the meals, classification criteria were found in several studies, which valued foods with an ED above 2.5 kcal/g or 4 kcal/g as “high energy density foods” and an ED ≤ 1.5 kcal/g indicated “low energy density foods”, while an ED < 0.6 kcal/g was defined as “very low ED” [16,17]. Based on this categorization, the ED of the investigated meals (0.70–1.02 kcal/g) can be estimated as low, irrespective of the portion type.

The German National Food Consumption Study (NVS, 1985–1988) and the EsKiMo study (2006) on dietary habits of children and adolescents in Germany demonstrated a decrease in the total fat intake during the last decades, which was also due to a reduced consumption of fats and oils, as well as fish, eggs and meat [23]. These observations are consistent with the results of the present study, showing a predominance of ovo-lacto-vegetarian meals in the school menus as well as a total fat content that fulfilled or tended to fall below the DGE/ÖGE reference values. Nonetheless, the quality and composition of the fat in school meals are crucial for pupils’ health. The fatty acid profile of food affects the fat metabolism in the body [24]. The composition of dietary fatty acids SFAs, MUFAs and PUFAs appears to influence hunger and satiety, which in turn are regulated by pancreatic and gut hormones [25]. It was found that a diet high in MUFAs and PUFAs was associated with a reduction in obesity [25]. In addition, the *n*-6 fatty acids and the *n*-6/*n*-3 PUFA ratio in phospholipids of blood cell membranes seem to play a role in obesity development and an increase in pro-inflammatory processes [26]. Given that common current diets exhibit high *n*-6/*n*-3 PUFA ratios of 20:1 or higher, the ratios identified for lunch meals in this study, between 2.6 and 4.1, were notably lower and also below the current *n*-6/*n*-3 PUFA ratios of 15 observed in the UK and Northern Europe [26]. Therefore, although PUFA levels were lower than MUFAs and SFAs in the fatty acid profile of the school meals in the present study, it can be assumed that the identified *n*-6/*n*-3 PUFA ratios indicate a favorable fat composition with the potential to prevent obesity in children and adolescents. Overall, these initial findings indicate that the QST objectives of health promotion in pupils and sustainability are given consideration by the study’s participating meal providers.

Nevertheless, different nutrients such as protein, sugar, vitamin C or salt showed discrepancies between the actual meal or food component content and the DGE/ÖGE reference values, requirements of the QST or the recommendations of the WHO.

In children and adolescents, the range of protein intake according to the DGE/ÖGE reference values includes both maintenance and growth requirements and therefore decreases with age [9]. The recommended protein energy percentage of total meal energy for pupils (7 to 19 years) is 6 to 10 E% [27]. This was clearly exceeded in the present study’s meals. The elevated protein content of the school meals was primarily attributable to the use of high-protein components, which constituted a quarter of the total number of meal components, and were derived from both animal and ovo-lacto-vegetarian sources. Compared to components of animal origin, ovo-lacto-vegetarian products have lower energy content. Therefore, a more frequent use of ovo-lacto-vegetarian components in menus could reduce energy intake and increase ingestion of additional desirable ingredients such as vitamins, minerals, dietary fiber or phytochemicals, but might deliver less protein quality. Nonetheless, a high protein intake in children and adolescents is discussed to have adverse health effects [27,28]. This may be due to the storage of protein energy in excess of that required for growth or early onset of puberty [28]. However, it should be noted that studies of protein exposure have primarily included younger children below the school age [27,28]. In addition, increasing the proportion of plant-based foods compared to animal-based foods not only contributes to a healthier diet but also reduces greenhouse gas emissions and the use of water resources [8,29].

Several studies demonstrated sugar as a risk factor for the development of obesity and related metabolic diseases, such as type 2 diabetes mellitus or dental caries in children, adolescents and adults [30,31,32]. To assess the sugar content of school meals according to recommendations of the WHO and the consensus paper of the DAG, the DDG and the DGE/ÖGE, only the total sugar content could be considered, as the analyses in the present study covered the total sugar content and did not distinguish between added sugars and sugars naturally present in the food components [11,20]. Furthermore, there is currently no established quantitative recommendation for total sugar intake for children and adolescents. The recommendations put forth by the WHO and the consensus paper of the DAG, the DDG and the DGE/ÖGE are limited to a maximum food content for free sugars (10 E%), which were employed in the present study to evaluate the total sugar content of the school meals [11,20]. Although this strategy results in a more rigorous interpretation of the permitted amount of sugar in school meals, it is aligned with the WHO’s objective of further reducing the free sugar content of foods to below 5% of the total energy [11,20]. Our study data analysis revealed that the majority of meals exceeded the maximum recommended intake of sugar. Similar outcomes were identified by Fox et al. (2021) in their examination of added sugars in school lunch meals in accordance with the Dietary Guidelines for Americans (DGA), utilizing data from 1207 school lunches in the School Nutrition and Meal Cost Study [33]. The processing of detailed food descriptions, recipes, portion size and number of servings prepared revealed that 69% of lunches exceeded the added sugar limit recommended by the DGA (<10% of total calories) [33].

Analysis of the sugar profile revealed fructose, sucrose and glucose as the most frequent sugars contained in the present meals. In the literature, fructose has been described, amongst others, as a major effector in the development of hypertension, insulin resistance and the induction of triglyceride synthesis and fat storage in the liver in children and adults [30,34,35]. Furthermore, Lustig et al. (2016) described children with consumption of sugar > 15 E% and fructose > 5 E% of the total energy intake as “high habitual sugar consumers” [35], which was true for both sugar and fructose content, at least within the lunch portion of the meals in the present study. Hence, in the future, meal providers should pay special attention to the controlled addition of sugars to meals, sensitize employees in this regard and reconsider the use and amount of convenience products such as ready-made marinades or desserts and prefer the usage of natural foods, such as fruits [8].

However, it should be noted that recent studies highlighted some inconsistencies in the reported adverse health effects of sugar, with a particular focus on the distinction between the effects of liquid (beverages) and solid food sugar content [36,37]. The present study exclusively considered solid foods since consumption of sugar-sweetened beverages at lunchtime is not recommended in the QST and were therefore not included in menus. Further investigations of the sugar content of school meals may benefit from a separate analysis of liquid and solid sugar-containing components, which could facilitate the derivation of health effects.

Amongst others, vitamin C plays a central role in the stimulation or modulation of the immune response, in bone mineral density and in neurological health and vitality [38]. In the present study, in most school meals, vitamin C was not detectable, thus DGE/ÖGE recommendations for vitamin C could not be fulfilled. Thereby, the vitamin C content variability of meal components could not be attributed to differences in portion size. It can be assumed that meals and/or individual components were subject to different preparation, transport or serving processes, which could include variations in cooking or transport time or in the length of serving time at the school. As the sample collections for the study were conducted at the final serving time, the resulting nutrient contents represent the component level at the maximum storage time. Water-soluble vitamins are, amongst others, sensitive to temperature, oxygen or light, and therefore vitamin C is prone to degrade during the different food processing steps [39]. Hence, the availability of fresh food, in conjunction with gentle cooking and preparation techniques, has the potential to enhance the supply of sensitive nutrients [8]. Meal providers, if possible, are requested to optimize food processing steps, transport routes and storage conditions in the future to ensure that sensitive nutrients such as vitamin C are preserved [8]. In addition to the naturally occurring vitamin C in food, ascorbic acid is also used as a synthetic food preservative [40]. The observed vitamin C contents in the present study may have been influenced by the addition of synthetic ascorbic acid. This may be particularly applicable to the use of convenience foods such as “Jelly and vanilla sauce” or “apple puree”.

Although sodium is an essential nutrient crucial for the maintenance of cellular homeostasis or fluid balance, several studies suggest that sodium intake affects blood pressure and blood pressure-related diseases, such as coronary heart disease or stroke [41,42]. Therefore, the WHO recommends a reduction in sodium intake to a maximum of 2 g sodium (<5 g salt) per day for adults—and for children, intake should be even less [43]. Of note, with salt contents > 3 g/portion in both analyzed portion types and sample collections (T1, T2), the meals already showed high salt/sodium values, exceeding recommendations (on the basis of sodium) adapted for lunch meal proportions [7,8]. In accordance with this, the salt content of more than half of the analyzed meal components exceeded the recommended benchmarks set forth by the WHO for food components [22]. This was probably due to the high salt content of ready-made products and the addition of salt by catering staff themselves to components such as pasta, rice or potatoes. These observations illustrate the necessity for a more attentive selection of foods and salt usage by the school meal providers.

Not least, the positive response to sugar and salt generally exists from birth, and taste preferences are formed early in life [44]. Therefore, high salt and/or sugar contents of school lunch meals might also impact students’ future taste and food preferences. A primary school menu low in salt, sugar and saturated fat was assumed as a sustainable diet that contributes to reducing greenhouse gas emissions, but taking into account recommendations for micronutrient and dietary fiber meal content in order to promote health [45]. These observations show that critically assessing the composition of school meals from a healthy but also sustainable perspective remains a challenge for all those involved in the nutrition of children and adolescents at school. On the one hand, a careful catering management of school meal providers should involve menu planning according to the recommendations of the QST on health promotion and sustainability [8]. This particularly includes a regional and seasonal choice of high-quality foods, consideration of students’ (and school employees’) tastes and wishes and the reduction of food waste by screening food waste amounts and adaptation of meal offers and portion sizes [8]. On the other hand, it should be noted that providing children with healthy nutrition should extend beyond the school environment to private households and is a key responsibility for parents and other caregivers.

### Strengths and Limitations of the Study

Given that most published studies on nutritional intake of children and adolescents are based on dietary questionnaires or interviews, this study presents rarely published laboratory data of nutrient analyses in school meals. The dataset includes a diverse range of school meals, comprising both primary and secondary portions, with a multitude of food component compositions. It must be taken into account that the sample of schools selected for the study was limited to those in the Thuringian project. Consequently, the results may serve as an illustrative example but cannot be assumed to be representative of school meals in other German federal states or Germany as a whole. Moreover, the present study focused on the composition of a mixed diet. Therefore, it is not possible to draw any conclusions on an ovo-lacto-vegetarian diet.

The secondary school count in the study and the resulting number of meal analyses were relatively low in both sample collections compared to primary schools. This resulted in larger CI (95%) ranges for energy values and different nutrients in the lunch meals in the secondary compared to the primary portion samples. Therefore, conclusions drawn from this part of the study cohort should be treated with caution, and further analyses involving larger sample sizes are required to reach more reliable conclusions in this regard. The requirements of the QST on frequency of food groups, such as whole grains or vegetables for a five-day protocol, could not be addressed by the sample collection of the present study. It is noteworthy that in accordance with the QST, the group of grain/grain products/potatoes and vegetables/salad appeared with the highest frequency, followed by dairy products and fruits. Nevertheless, the results of a detailed and long-term school menu analysis will be published elsewhere. Furthermore, the study did not evaluate the selection frequency of the meals analyzed or the quantity consumed or discarded by students and school staff. It is therefore evident that our results represent the offer of major and minor nutrients, rather than reflecting the actual intake of the students. In the context of nutrient supply and sustainability, further studies are needed to address these issues in more detail.

## 5. Conclusions

The present study demonstrates that children and adolescents at the investigated Thuringian schools are generally well supplied with most of the basic nutrients recommended for the portion of lunch meals, thereby supporting healthy development and preventing nutrition-related diseases. Nevertheless, some critical nutrients need to be given greater focus to further improve meal quality in accordance with the QST. The implementation of a deepened and continuous control of meal plans regarding sugar and salt origins in school meals could help to reduce excessive intake of ingredients with adverse health effects. Moreover, an improved tracking of food component processing is necessary to ensure sufficient intake of vulnerable nutrients such as vitamin C. This might include, amongst others, a detailed check of individual meal recipes and food components, esp. for convenient products, complemented by the communication and sensitization of the kitchen staff and process standardization.

## Figures and Tables

**Table 1:** Descriptive characteristics of the study.

Characteristic	Timepoint 1 (T1)	Timepoint 2 (T2)
Duration	September 2020–November 2021	January 2022– December 2022
General school count	26	25
Count of primary schools ^1^	12	11
Count of comprehensive schools ^2^	11	10
Count of secondary schools ^3^	3	4
Analyzed single components	96	88
Calculated meals	25 ^4^	24 ^4^
Thereof: calculated meals for primary school level	23 ^5^	21 ^6^
Thereof: calculated meals for secondary school level	4 ^5^	8 ^6^
Types of food preparation/serving		
Cook & Serve	3	3
Cook & Hold	18	14
Mixed Cook & Serve/Cook & Hold	4	7

^1^ First to 4th grade. ^2^ First to 10th/12th grade. ^3^ Fifth to 10th/12th grade. ^4^ Two schools used one common canteen with the same offer. ^5^ Two meals were collected in one school, respectively. These schools were subdivided into primary and secondary school levels due to discrimination between primary and secondary pupils during food delivery (different amounts of food were served). ^6^ Five meals were collected in one school, respectively. These schools were subdivided into primary and secondary school levels due to discrimination between primary and secondary pupils during food delivery (different amounts of food were served).

**Table 2:** Mean energy content and energy density of analyzed school meals.

Portion Type	Timepoint	*n*	Mean ± SD	CI [95%]
			Energy [kcal/portion]	
Primary	T1	23	444.5 ± 131.5	387.6–501.3
Secondary	T1	4	495.9 ± 130.5	288.3–703.5
Primary	T2	21	453.1 ± 185.9	368.5–537.7
Secondary	T2	8	626.9 ± 380.9	308.5–945.3
			Energy density [kcal/g]	
Primary	T1	23	0.8 ± 0.2	0.7–0.9
Secondary	T1	4	0.7 ± 0.2	0.4–1.1
Primary	T2	21	0.8 ± 0.3	0.7–1.0
Secondary	T2	8	0.9 ± 0.3	0.7–1.1

Primary portion: ≥7 to <10 years, secondary portion: ≥10 to 19 years, CI: confidence interval, SD: standard deviation.

**Table 3:** Food components of school meals with high energy density at both timepoints.

Food	Timepoint	Energy Density [kcal/g]	Portion Size
Grated cheese *	T1	3.0	Primary
Grated cheese *	T1	3.4	Primary
Grated cheese *	T1	3.3	Primary
Roll (whole grain)	T1	2.5	Primary
Fried sausage	T1	2.9	Primary
Butter	T1	7.5	Primary
Roll (wheat)	T1	2.5	Primary
Fried sausage (turkey)	T2	3.1	Primary
Pork escalope	T2	2.6	Secondary

* Samples were collected in different schools.

**Table 4:** Results of main nutrient contents of school lunch meals.

Nutrient	*n*	Timepoint	Mean ± SD	CI (95%)	Reference Value [g/Lunch Meal]
Primary portion					
Carbohydrates [g]	23	T1	57.3 ± 19.8	48.8–65.9	50.0
	21	T2	53.4 ± 17.1	45.7–61.2	
Fat [g]	23	T1	12.1 ± 8.0	8.6–15.5	13.3
	21	T2	15.7 ± 14.6	9.1–22.4	
Protein [g]	23	T1	21.6 ± 9.4	17.5–25.6	6.5
	21	T2	19.4 ± 9.5	15.1–23.7	
Dietary fiber [g]	23	T1	10.2 ± 5.6	7.8–12.7	7.5 ^#^
	21	T2	10.2 ± 3.6	8.6–11.9	
Secondary portion					
Carbohydrates [g]	4	T1	60.2 ± 9.6	44.9–75.5	64.6
	8	T2	68.9 ± 22.6	50.1–87.8	
Fat [g]	4	T1	16.2 ± 10.0	0.2–32.2	17.2
	8	T2	24.8 ± 28.2	1.2–48.3	
Protein [g]	4	T1	22.4 ± 10.0	6.5–38.2	11.8
	8	T2	25.4 ± 16.0	12.1–38.7	
Dietary fiber [g]	4	T1	10.0 ± 3.8	4.0–16.0	7.5 ^#^
	8	T2	13.2 ± 4.1	9.8–16.7	

Primary portion: ≥7 to <10 years, secondary portion: ≥10 to 19 years; references values are calculated from [7,8] (for details, see chapter data analysis). CI: confidence interval, SD: standard deviation, ^#^: DGE/ÖGE reference values for children and adolescents were not available; adult reference values were used.

**Table 5:** Classification of sugar content in individual meal components, as well as entire school meals, according to current recommendations for maximum free sugar content [11].

Total Sugar Content > 10 E%	Timepoint	
	T1	T2
Components primary portions *	18.3%	16.0%
Components secondary portions *	11.5%	12.3%
Meals primary portions *	88.9%	86.2%
Meals secondary portions *	74.1%	72.4%

Primary portion: ≥7 to <10 years, secondary portion: ≥10 to 19 years. *: Total sugar content for primary and secondary portions of lunch meals, calculated according to current recommendations for a maximum of 10 E% of free sugars on total energy intake. T1—timepoint 1, T2—timepoint 2.

**Table 6:** Results of vitamin contents of school lunch meals.

Vitamin	*n*	Timepoint	Mean ± SD	CI (95%)	Reference Value [g/Lunch Meal]
Primary portion					
Vitamin C [mg]	23	T1	14.1 ± 34.4	N.D.	11.3
	21	T2	6.8 ± 19.2	N.D.	
Vitamin E [mg]	23	T1	3.1 ± 1.8	2.3–3.9	2.3
	21	T2	4.7 ± 3.4	3.2–6.3	
Vitamin B_1_ [mg]	23	T1	0.2 ± 0.1	0.1–0.2	0.2
	21	T2	0.2 ± 0.2	0.1–0.3	
Folate [µg]	23	T1	34.5 ± 19.1	26.2–42.7	45.0
	21	T2	43.6 ± 39.1	25.8–61.3	
Secondary portion					
Vitamin C [mg]	4	T1	13.0 ± 16.4	N.D.	26.3
	8	T2	4.7 ± 8.4	N.D.	
Vitamin E [mg]	4	T1	5.3 ± 2.6	1.1–9.4	3.8
	8	T2	6.5 ± 5.2	2.2–10.8	
Vitamin B_1_ [mg]	4	T1	0.2 ± 0.1	0.0–0.3	0.4
	8	T2	0.3 ± 0.2	0.1–0.4	
Folate [µg]	4	T1	53.3 ± 25.9	12.0–94.5	75.0
	8	T2	58.5 ± 45.1	20.7–96.2	

Primary portion: ≥7 to <10 years, secondary portion: ≥10 to 19 years, CI: confidence interval, SD: standard deviation, N.D.: not detectable—due to low detectable vitamin C contents, CI calculation was refused; reference values are calculated from [7,8] (for details, see chapter data analysis).

**Table 7:** Vitamin C content of foods with detectable vitamin C contents.

Food	Vitamin C Content [mg/100 g Portion] *	Food	Vitamin C Content [mg/100 g Portion] *
Timepoint T1		Timepoint T2	
Apple puree	69	Apple curd	5
Blueberry curd	2	Jelly and vanilla sauce	48
Chinese cabbage with mandarin	7	Paprika, tomato ^#^	40
Green cabbage salad with cream	34	Potato ^#^	10
Kiwi	147	Potato ^#^	3
Paprika, tomato ^#^	15	Vegetable mix (carrots, cauliflower, broccoli)	3
Potato ^#^	6	Vegetable mix (Romanesco, carrot, paprika, corn) in white sauce	5
Salad (green cabbage, iceberg, yogurt dressing)	91	Yogurt with berries	6
Tomato salad	3		

* Only values of the primary portion were considered; ^#^ samples were collected in different schools.

**Table 8:** Results of mineral contents of school lunch meals.

Nutrient	*n*	Time-Point	Mean ± SD	CI (95%)	Reference Value [mg/Lunch Meal]
Primary portion					
Calcium [mg]	23	T1	149.0 ± 94.6	108.1–189.9	225.0
	21	T2	188.8 ± 161.2	115.4–262.1	
Iron [mg]	23	T1	3.7 ± 2.6	2.5–4.8	2.5
	21	T2	3.1 ± 1.9	2.3–4.0	
Magnesium [mg]	23	T1	74.5 ± 20.9	65.4–83.5	57.5
	21	T2	77.8 ± 28.3	64.9–90.7	
Sodium [mg]	23	T1	1382.6 ± 477.8	1176.0–1589.3	187.5
	21	T2	1587.9 ± 550.9	1337.1–1838.7	
Secondary portion					
Calcium [mg]	4	T1	253.4 ± 137.5	34.5–472.2	300.0
	8	T2	395.0 ± 377.7	79.2–710.8	
Iron [mg]	4	T1	2.5 ± 1.0	0.9–4.1	3.8
	8	T2	4.3 ± 3.7	1.2–7.4	
Magnesium [mg]	4	T1	91.3 ± 32.3	40.0–142.7	82.5
	8	T2	110.0 ± 49.1	68.9–151.0	
Sodium [mg]	4	T1	1749.6 ± 295.3	1279.8–2219.5	375.0
	8	T2	1657.7 ± 855.7	942.3–2373.1	

Primary portion: ≥7 to <10 years, secondary portion: ≥10 to 19 years, CI: confidence interval, SD: standard deviation. Reference values are calculated from [7,8] according to the “quarter approach” (for details, see chapter data analysis).

**Table 9:** Count of school meal components exceeding WHO salt benchmarks, as well as WHO salt benchmarks with tolerance range plus 10%, 20% and 50% at both timepoints [22].

Category of WHO Salt Benchmark [mg/100 g Food]	Timepoint	% Components >WHO Benchmark	Total Component Count
WHO benchmark	T1	63	104
WHO benchmark +10%	T1	63	
WHO benchmark +20%	T1	62	
WHO benchmark +50%	T1	51	
WHO benchmark	T2	70	106
WHO benchmark +10%	T2	65	
WHO benchmark +20%	T2	62	
WHO benchmark +50%	T2	58	

WHO—World Health Organization.

## Data Availability

The data presented in the study are available upon reasonable request from the corresponding author.

## Abbreviations

The following abbreviations are used in this manuscript:

CI	Confidence interval
DAG	Deutsche Adipositas-Gesellschaft e.V., German Obesity Society
DDG	Deutsche Diabetes Gesellschaft e.V., German Diabetes Society
DGA	Dietary Guidelines for Americans
DGE	Deutsche Gesellschaft für Ernährung e.V./German Nutrition Society
E%	Energy percentage
ED	Energy density
MUFAs	Monounsaturated fatty acids
ÖGE	Österreichische Gesellschaft für Ernährung/Austrian Nutrition Society
PUFAs	Polyunsaturated fatty acids
QST	DGE-Qualitätsstandard für die Verpflegung in Schulen
SD	Standard Deviation
SFAs	Saturated fatty acids
T1, T2	Timepoint 1, Timepoint 2
TSMP	Teilsubventionierungsprojekt der Mittagsmahlzeiten zur Qualitätsverbesserung der Speisenversorgung an Thüringer Schulen
WHO	World Health Organization
